# Bilateral ureteropelvic disruption following blunt abdominal trauma: Case report

**DOI:** 10.1186/1471-2490-11-14

**Published:** 2011-07-07

**Authors:** Fumiaki Iwase, Yoshibumi Miyazaki, Tastuho Kobayashi, Hiroko Kikuchi, Kiyoshi Mastuda

**Affiliations:** 1 Emergency and Critical Care Medical Centre, Yamanashi Prefectural Central Hospital, 1-1-1 Fujimi, Kofu, Yamanashi 400-8506, Japan

## Abstract

**Background:**

Ureteral injury occurs in less than 1% of blunt abdominal trauma cases, partly because the ureters are relatively well protected in the retroperitoneum. Bilateral ureteral injury is extremely rare, with only 10 previously reported cases. Diagnosis may be delayed if ureteric injury is not suspected, and delay of 36 hours or longer has been observed in more than 50% of patients with ureteric injury following abdominal trauma, leading to increased morbidity.

**Case presentation:**

A 29-year-old man was involved in a highway motor vehicle collision and was ejected from the front passenger seat even though wearing a seatbelt. He was in a preshock state at the scene of the accident. An intravenous line and left thoracic drain were inserted, and he was transported to our hospital by helicopter. Whole-body, contrast-enhanced computed tomography (CT) scan showed left diaphragmatic disruption, splenic injury, and a grade I injury to the left kidney with a retroperitoneal haematoma. He underwent emergency laparotomy. The left diaphragmatic and splenic injuries were repaired. Although a retroperitoneal haematoma was observed, his renal injury was treated conservatively because the haematoma was not expanding. In the intensive care unit, the patient's haemodynamic state was stable, but there was no urinary output for 9 hours after surgery. Anuresis prompted a review of the abdominal x-ray which had been performed after the contrast-enhanced CT. Leakage of contrast material from the ureteropelvic junctions was detected, and review of the repeat CT scan revealed contrast retention in the perirenal retroperitoneum bilaterally. He underwent cystoscopy and bilateral retrograde pyelography, which showed bilateral complete ureteral disruption, preventing placement of ureteral stents. Diagnostic laparotomy revealed complete disruption of the ureteropelvic junctions bilaterally. Double-J ureteral stents were placed bilaterally and ureteropelvic anastomoses were performed. The patient's postoperative progress was satisfactory and he was discharged on the 23^rd ^day.

**Conclusion:**

Diagnosis of ureteral injury was delayed, although delayed phase contrast-enhanced CT and abdominal x-rays performed after CT revealed the diagnosis early. Prompt detection and early repair prevented permanent renal damage and the necessity for nephrectomy.

## Background

Ureteral injury occurs in less than 1% of abdominal trauma cases [1,2]. Bilateral ureteral injury is extremely rare, with only 10 previously reported cases [3-6]. Although ureteral injuries are often missed because of haemodynamic instability and other concomitant injuries, early diagnosis is important to prevent complications. We report a case of bilateral complete ureteropelvic disruption secondary to blunt trauma that was suspected on viewing abdominal x-rays performed after contrast-enhanced computed tomography (CT). Diagnosis was confirmed early and the injury was repaired promptly without any complications.

## Case presentation

A 29-year-old man was involved in a highway motor vehicle collision and was ejected from the front passenger seat even though wearing a seatbelt. He was in a preshock state at the scene of the accident. He complained of dyspnea and had decreased breath sounds on the left side of his chest. An intravenous line and left thoracic drain were inserted at the scene, and he was transported to our hospital by helicopter. A wide seatbelt mark was noted on his abdomen. Whole-body, contrast-enhanced CT scan showed left diaphragmatic disruption, splenic injury, and a grade I injury to the left kidney with a retroperitoneal haematoma. He underwent emergency laparotomy. The rectus muscle was torn and a massive haemoperitoneum was observed. The left diaphragmatic and splenic injuries were sutured and the transverse mesocolon was repaired. The renal injury was treated conservatively because the retroperitoneal haematoma was not expanding. Ten units of packed red blood cells were transfused during surgery, and the patient's haemoglobin level increased to 10.5 g/dL.

**Figure 1:**
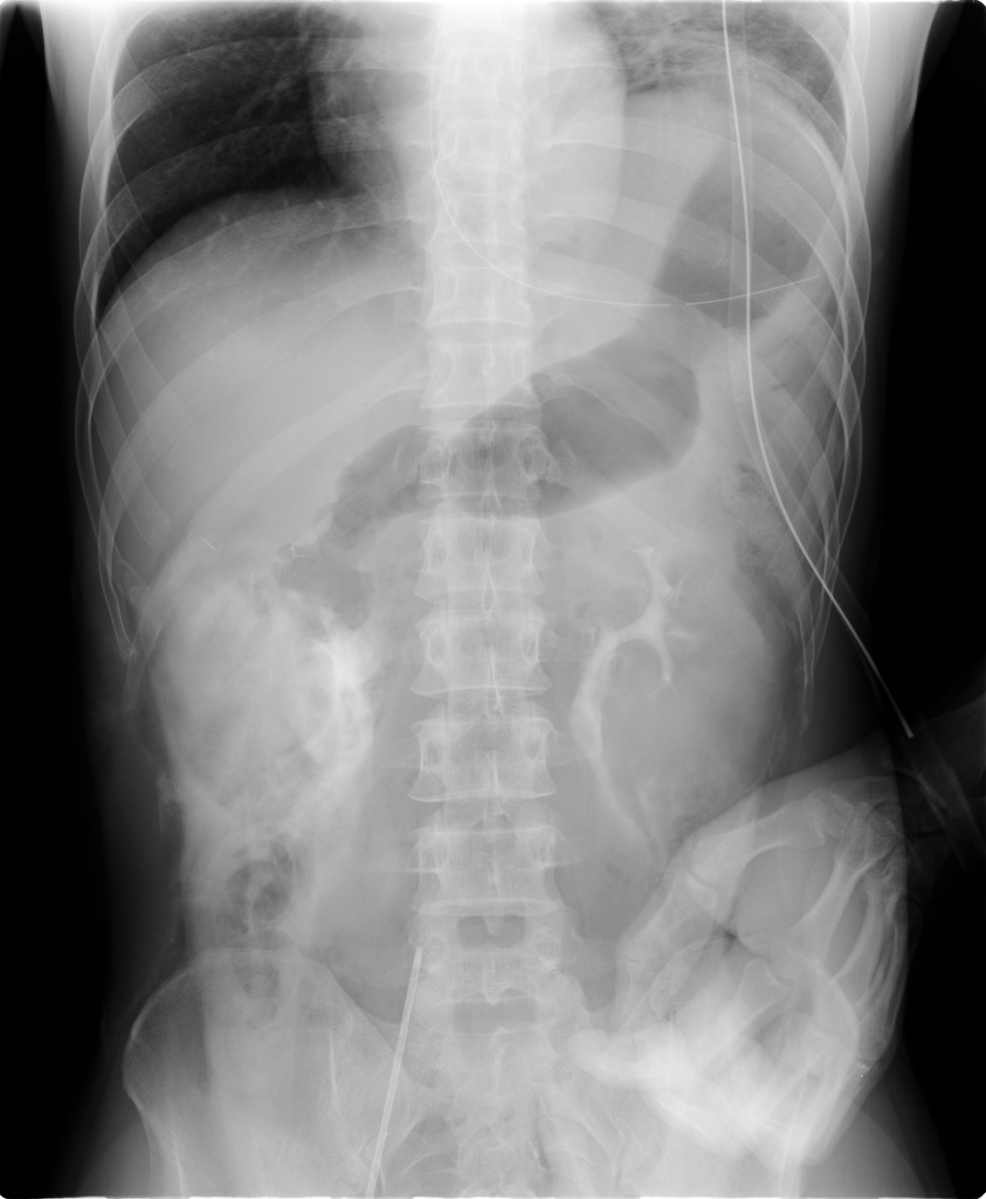
**Abdominal x-ray performed after contrast-enhanced CT on admission shows leakage of contrast material from the ureteropelvic junctions bilaterally**.

**Figure 2:**
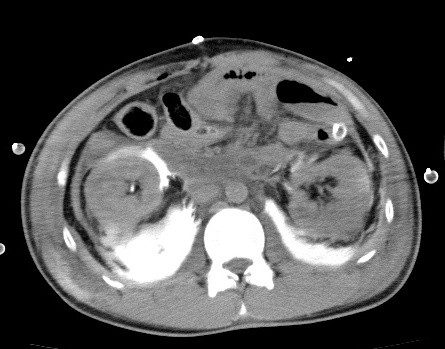
**Abdominal CT scan at 9 hours after surgery shows contrast retention in the perirenal retroperitoneum bilaterally**.

In the intensive care unit, the patient's blood pressure was 110/60 mmHg and his heart rate was 106 beats per minute, but no urinary output was observed for 9 hours after surgery. Anuresis prompted a review of the abdominal x-ray (Figure 1) which had been performed after the contrast-enhanced CT. Leakage of contrast material from the ureteropelvic junction was detected, and review of the CT scan revealed contrast retention in the perirenal retroperitoneum bilaterally (Figure 2). He underwent cystoscopy and bilateral retrograde pyelography, which showed bilateral complete ureteral disruption, preventing placement of ureteral stents. Diagnostic laparotomy revealed complete disruption of the ureteropelvic junctions bilaterally. Double-J ureteral stents were placed bilaterally and ureteropelvic anastomoses were performed. The patient's postoperative progress was satisfactory and he was discharged on the 23^rd ^day.

## Discussion

Ureteral injuries resulting from blunt trauma are uncommon and represent less than 1% of all traumatic genitourinary injuries [2]. Bilateral ureteral injuries have only been previously reported in 10 cases [3-12]. According to the National Trauma Data Bank (2002-2006), ureteral injuries are seen in approximately 3 per 10,000 trauma admissions and occur less often in blunt trauma than in penetrating trauma [13].

Ureteral injury is often missed. A diagnostic delay of 36 hours or longer has been observed in more than 50% of patients [14]. Most delays occur secondary to haemodynamic instability and other injuries. Prompt detection and early repair is necessary to prevent nephrectomy, with delays leading to increased complication and and nephrectomy rates. McGinty *et al *described a nephrectomy rate of up to 32% in cases of delayed recognition compared with 4.5% in cases that were diagnosed early [15]. For early diagnosis in suspected cases of ureteral injury, either delayed-phase contrast-enhanced CT scan or abdominal x-ray following contrast-enhanced CT is recommended.

In this case, we missed the ureteral disruption until after the first surgery, and became suspicious due to postoperative anuria. We then detected the injuries by re-examining the abdominal x-ray performed after contrast-enhanced abdominopelvic CT. If we had overlooked his ureteric injuries, bilateral nephrectomy may have resulted. Retrograde pyelography is very sensitive and specific and is the reference standard for diagnostic imaging, but is often time-consuming and impractical in a patient who is unstable and/or has multiple injuries. Therefore, the excretory phase of a contrast-enhanced CT scan or an x-ray of the kidney, ureters and bladder after contrast-enhanced CT is recommended [16,17]. A high index of suspicion must be maintained because delayed diagnosis results in higher complication rates.

## Conclusions

Ureteral injures are frequently missed due to haemodynamic instability and other injuries. It is important that trauma specialists recognise additional injuries after major trauma to prevent complications.

## Competing interests

The authors declare that they have no competing interests.

## Authors' contributions

FI drafted the report, and approved the final version of the manuscript.

YM, TK, HK, and KM cared for the patient and approved the final version of the manuscript.

## Authors' information

Emergency and Critical Care Medical Centre, Yamanashi Prefectural Central Hospital, Kofu, Yamanashi 400-8506, Japan.

## Pre-publication history

The pre-publication history for this paper can be accessed here:

http://www.biomedcentral.com/1471-2490/11/14/prepub
